# Supplementary material for: Research on composite foundation rheology using the combined rheological element model
Source: PLoS One. 2025 Jul 10;20(7):e0327560. doi: 10.1371/journal.pone.0327560 (PMC12244545; doi:10.1371/journal.pone.0327560)
Supplement: Attachment — Submitted filename: review.pdf [file pone.0327560.s006.pdf]

The authors have made explicit changes and improvements to the paper in response to the reviewers' comments, as detailed below:

1. The authors have provided detailed maps and explanations of the experimental parameters and monitoring point layouts in the revised manuscript, supplemented by high-resolution relevant diagrams.
2. The authors clearly state the innovation of the paper in the introduction section.
3. The authors have optimized the structure of the paper, added a list of symbols and adjusted the title numbering to improve the logic and readability of the paper.
4. The authors have reorganized the content position of the model validation section to enhance the logic of the paper; and added a 2D COMSOL result chart and a model construction flow chart to further clarify the research process.
5. The authors have added a new section on research limitations, pointing out that the existing models do not sufficiently take into account anisotropy and multi-field coupling effects in practical applications, and suggesting directions for future research.
6. The authors have touched up the language of the article and fixes the terminology problem.

The revision of the article responds to the comments made by the reviewers, resulting in a significantly improved paper in terms of structure, content and scientific quality, which meets the requirements for publication, and a small amount of proofreading is recommended.
