# Supplementary material for: Research on composite foundation rheology using the combined rheological element model
Source: PLoS One. 2025 Jul 10;20(7):e0327560. doi: 10.1371/journal.pone.0327560 (PMC12244545; doi:10.1371/journal.pone.0327560)
Supplement: Attachment — Submitted filename: Response to Reviewers.docx [file pone.0327560.s005.docx]

Dear Ph.D. Ahmed M. Saqr and reviewers:

Thank you for your letter and the reviews’ comments on our manuscript entitled ”Research on composite foundation rheology using the combined rheological element model ” (PONE-D-24-13121).Those comments are very helpful for revising and improving our paper,as well as the important guiding significance to other research.We have studied the comments carefully and made corrections which we hope meet with approval.Firstly, the format of the manuscript was modified according to the style requirements of PLOS ONE, and the scholarship number was checked in the "funding information" section. At the same time, the supporting information file was uploaded. In addition, the abstract on the online submission form has been modified to make it consistent with the abstract in the manuscript, and the references have been organized and improved.This work was supported by the Natural Science Foundation of Shanxi Province[No.202103021224151], the Provincial Reform High-level Talents Introduction of Shanxi Agricultural University of China[Grant No.2021XG009], the National Natural Science Foundation of China[No. 42207102].

The main corrections are in the manuscript and the responds to the reviews’ comments are as follows.

Replies to the reviewers’ comments:

Reviewer #1:

1. The experimental parameters derived from Table 1, along with the basic information and results of the experiments, need to be provided.

Response:Due to the large number of calculation parameters used in this paper, combined with the concrete engineering characteristics of the composite foundation of Dakai clay core rockfill dam, the final parameters are mainly obtained through empirical values, indoor and outdoor tests and inverse calculation. Firstly, Poisson's ratio μ and foundation bearing capacity parameter β are related to soil type, compactness, water content, friction Angle and other factors, and cannot be directly obtained through experiments. Therefore, empirical values recommended in literature [26] are adopted. The permeability coefficient k, bulk density γ and porosity v were obtained by outdoor sampling combined with indoor tests. Secondly, by using the mathematical formula of the improved rheological model and combining with the settlement monitoring data of the dam body during the filling period, the initial elastic modulus E and E_1_, the elastic modulus E_0_ and E_2_ of the creep model and the viscosity coefficient η of the creep model are obtained through inversion calculation. In order to verify the rationality of the creep parameter values of the reinforced area and the underlying layer, To ensure the accuracy of the numerical model calculation results, the inversion calculation parameters were compared with the rheological experimental parameters of soil and stone in each layer, as shown in Table 3. The results show that the model parameters determined by inversion calculation are feasible. For details, see lines 295-305 and 316-325 of the manuscript.

2. The specific layout diagram of monitoring points needs to be incorporated.

Response:We have added the monitoring system layout in the revised draft uploaded, as shown in Fig 6.

3. It is necessary to slightly adjust the structure of the paper. Firstly, validate the rationality of this model, for example, by illustrating the differences between calculated displacement values and monitoring values. Utilizing a histogram might not provide the most intuitive representation; I suggest employing alternative methods. Following this, discuss the relevant patterns of change.

Response:As you suggested, we have put the content of model validation after the modeling and before the analysis of results . After the establishment of the model, through the verification of displacement, stress and seepage, the measured values are basically consistent with the calculated results of the model, which proves the effectiveness of the model. Then the dam displacement, stress and seepage analysis are carried out.This adjustment makes the article more logical.In addition, for the problem that the histogram expression in the model verification content is not intuitive, we have listed the comparison results between the model and the measured values in the form of a table, and attached the error value between the two, so that the results can be clearly presented.

4. Does the consolidation theory used here specifically refer to the Biot consolidation theory, or something else?

Response:Yes, the consolidation theory used in the paper is Biot consolidation theory, highlighted in line 342 of the manuscript.

Reviewer #2:

1. What is the novelty of this manuscript? It should be clearly mentioned in the last paragraph of the introduction.

Response:The novelty of the manuscript is that the coupling model of seepage and stress under the consolidation rheological effect of the dam foundation is established in view of the current situation that the influence of the rheological effect of the soft soil composite foundation on the clay core rockfill dam is still unclear, which makes up for the deficiency of previous studies that only consider the consolidation and settlement behavior of the foundation while ignoring the rheological effect. This paper provides a theoretical basis for the study of rheological model of soft soil composite foundation with deep cover layer, and takes the Dakai clay core rockfill dam project on soft soil composite foundation as an example to define the influence mechanism of rheological foundation on clay core rockfill dam on soft soil composite foundation.We have revised the last paragraph of the introduction in the revised manuscript uploaded, in lines 99-117 .

2. Please, replace all the figures with high-resolution ones.

Response:According to your suggestion, I have replaced all the pictures in the manuscript with high-resolution ones, see the revised draft uploaded for details.

3. You should add the limitation of your study at the end of the paper before the conclusion section.

Response:The composition of each layer of geotechnical materials in the foundation with deep cover layer is complex, and the non-uniformity of each layer of materials inside the foundation will make the geotechnical characteristics of obvious anisotropy. However, considering the solvable and computational accuracy of the model, the models proposed in this paper for the deep cover layer are all built on the transverse isotropic medium, which does not fully meet the love situation of the ground in the actual project. In the future study, the rheological properties of anisotropic rock and soil, especially the rheological effects under the coupling of seepage stress, still need to be further analyzed and studied.We have supplemented this part in lines 651-656 of the manuscript.

4. What is your recommendation for future studies to enhance the applied methodology? You can discuss how to relate your research findings with sustainable development goals (SDGs) to achieve more environmental, economic, and social benefits. References to cite:

https://doi.org/10.1016/j.ejrh.2024.101703

https://doi.org/10.1016/j.gsd.2024.101087

https://doi.org/10.1007/978-981-99-4101-8_27

https://doi.org/10.1007/978-981-99-1381-7_6

Response:In the future research, for the simulation of composite soft soil foundation, the influence of factors such as the thickness and location of each layer of soil on the upper and lower layers of soil can be considered, and these effects can be parameterized and introduced into the analysis and calculation of the model. In addition, the complex environment of actual engineering can also be considered. Rheological properties of composite soft soil foundation under multi-field coupling conditions, such as temperature, stress, seepage, chemical reaction, etc., are of guiding significance for dam construction in the deep cover layer of soft soil in coastal, inland waterway or lake area.

Yours sincerely,

Prof. Haitao Mao

Email addresses: maohaitao1234@163.com
