# Supplementary material for: Research on composite foundation rheology using the combined rheological element model
Source: PLoS One. 2025 Jul 10;20(7):e0327560. doi: 10.1371/journal.pone.0327560 (PMC12244545; doi:10.1371/journal.pone.0327560)
Supplement: Attachment — Submitted filename: Response_to_Reviewers_auresp_2.docx [file pone.0327560.s007.docx]

Dear academic editor Qiang Li and reviewers:

Thank you for your letter and the reviews’ comments on our manuscript entitled “Research on composite foundation rheology using the combined rheological element model ” (PONE-D-24-13121).Those comments are very helpful for revising and improving our paper,as well as the important guiding significance to other research.We have studied the comments carefully and made corrections which we hope meet with approval.The main corrections are in the manuscript and the responds to the reviews’ comments are as follows.

Replies to the reviewers’ comments:

Reviewer #3:

1. The high number of parameters employed (see Table 1) requires a "list of symbols" section where the readers can easily access all the definitions.

Response:According to your suggestion, we have sorted out all the parameter symbols in the manuscript, and modified some places (for example, the same symbols were used to express different parameters), and the corresponding highlights were also made in the manuscript. Our list explains the meaning of each parameter symbol, along with the corresponding units, and finally places the symbol list after Conclusion, specifically at line 556 in the revised manuscript.

2. To improve the readability and logic of this text, please precede all titles of the manuscript with serial numbers, such as "1, 2, 1.1, 2.1...".

Response:According to your suggestion, we have numbered the main body of the manuscript. The first, second and third headings are denoted by 1, 1.1 and 1.1.1 respectively. The specific changes have been highlighted in yellow in the revised manuscript.

3. The theoretical formulas (1)-(11) of the numerical model need to be given in the manuscript with corresponding references.

Response:According to your suggestion, we have added references to formulas (1) - (4), specifically see references [24] and [27-29]. Formula (4) is summarized according to the research ideas in the literature rather than directly citing the formula in the literature [29], and mainly expresses the total stress and total strain of the fractional merchant model.In addition, on the basis of formula (4), formulas (5) - (10) are derived by Laplace transform and inverse transformation, and formula (11) is the boundary condition given to the rheological model of composite foundation, without citing relevant literature. At the same time, thank you very much for asking this question, because in the process of replying to this question, we found some writing errors in the formula and corrected them in time, please see the revised manuscript for details.

4. Add a meshing diagram of the dam model in the manuscript in COMSOL Multiphysics.

Response:According to your suggestion, we have added the grid diagram of the dam model in the revised manuscript, as shown in Figure 8 of lines 322-323.

5. The title "Modeling" on line 350 is necessary to specify which model it refers to, the existing title makes the logical framework of the article very confusing. The narrative in this section may be described in the form of a flowchart.

Response:According to your suggestion, we deleted the titles 1, 2, and 3 in the modeling section, and adjusted the text expression of these parts to make the structure consistent with the full text. At the same time, the flow chart generated according to the model building steps is also added to show the model generation process more clearly. See Figure 7 on lines 322-323 for details.

6. For the model validation part, it is recommended to provide a 2D result diagram in COMSOL Multiphysics in the manuscript, which can more intuitively reflect the accuracy of the model.

Response:According to your suggestion, we have retrieved the point result graphs of measuring points DK1, DK2, SW1, SW2, SG1 and SG2 in COMSOL, so as to intuitively reflect the correctness of the model. For details, see Fig 9 and Fig 10 in the revised manuscript.

7. It is necessary to improve the resolution of the picture, such as Figure 11, etc., the display of dense streamlines is not clear. It is recommended that the results in the text be represented by color cloud plots, discarding the contour plots. Simple contour plots are not conducive to analyzing the results.

Response:According to your suggestion, we have changed all the contour maps of horizontal displacement, settlement, stress and seepage field in the manuscript into color cloud maps. The analysis of the results in the original manuscript was completed on the basis of contour maps. After changing to cloud maps, we re-analyzed the results of the cloud maps, so the text expression of this part is somewhat different from the original one. See line 400-419, 428-443, 455-477 and 484-500 for details.

8.“Comsol” should be “COMSOL”

Response: We checked the manuscript for typos regarding Comsol and changed all Comsol to COMSOL, see lines 11, 80, 296, and 331 for details.

9. Please check and revise the text for language problems.

Response:According to your suggestions, we have carefully checked the language problems in the manuscript and made revisions, which have been highlighted in green in the revised manuscript. At the same time, if you feel that the manual correction has not met the requirements of the paper publication, we can further polish the manuscript in the later stage.

Yours sincerely,

Prof. Haitao Mao

Email addresses: maohaitao1234@163.com
