# Supplementary material for: Research on composite foundation rheology using the combined rheological element model
Source: PLoS One. 2025 Jul 10;20(7):e0327560. doi: 10.1371/journal.pone.0327560 (PMC12244545; doi:10.1371/journal.pone.0327560)
Supplement: Attachment — Submitted filename: Response_to_Reviewers_auresp_3.docx [file pone.0327560.s008.docx]

Dear academic editor and reviewers:

Thank you for your letter and the reviews’ comments on our manuscript entitled “Research on composite foundation rheology using the combined rheological element model ” (PONE-D-24-13121).Those comments are very helpful for revising and improving our paper,as well as the important guiding significance to other research.We have studied the comments carefully and made corrections which we hope meet with approval.The main corrections are in the revised manuscript and the responds to the reviews’ comments are as follows.

Replies to the reviewers’ comments:

Reviewer #3:

Although this manuscript has undergone extensive revisions by the authors, the issues within it still raise significant concerns for me. In fact, the depth of the article is far from adequate. Many expressions and figures are also not presented according to standards. After careful consideration, I firmly believe that this manuscript is not suitable for publication. The organization of the article is indeed lacking. The research methods, particularly the mathematical modeling section, appear quite chaotic and lack clarity. Additionally, the validation part is also questionable; it is unclear whether the monitoring data, such as displacement, was obtained accurately, and the manuscript does not provide detailed explanations, merely comparing the data with simulation results. The results and discussion section is also insufficiently thorough.

Response: First of all, I would like to express my sincere appreciation for the time and effort you have invested in reviewing our manuscript. We value the valuable comments you have provided and have carefully considered each piece of feedback.

1. Regarding the problem that the depth of the article is far from enough: In previous studies on the calculation of composite foundation models, only the consolidation and settlement behavior of the foundation is usually considered, while the rheological effect has a very significant influence on the stability and settlement of the foundation. Therefore, on the basis of the classical combined model of composite foundation, we established a coupled model of seepage and stress under the consolidation rheological effect of soft soil composite dam foundation, and took the Dakai clay core wall rockfill dam project on soft soil composite foundation as an example, established the calculation model and compared the observed and simulated values with the actual observation data. It is found that the difference between the established model and the observation results is not significant, which verifies the accuracy of the established model, so we believe that this manuscript provides a certain theoretical basis for the study of the rheological modeling of soft soil composite foundations with deep cover.
2. Regarding the problem of non-standard expressions and graphs: In the first revision, we realized the problem of non-standard mathematical expressions in the manuscript, so in the last round of revision, we reviewed the formulae for writing and labeling errors, and added a list of symbols after the conclusions to explain the meanings of all symbols appearing in the formulae of the manuscript, so that the readers can understand the meanings expressed by the formulae more clearly. As for the issue of substandard graphs, we apologize that we are not quite sure exactly what aspect you are referring to. Figures 1-3 are the descriptions of the model, and Figures 4-8 are the project location map, dam filling materials, dam foundation treatment measures, dam monitoring system layout schematic and dam monitoring data acquisition flow chart, respectively. According to your suggestions, we have further adjusted the clarity of the pictures, please see the revised manuscript with track changes for details. In the manuscript, the change diagrams (i.e., Fig.10-Fig.16) about the grid division, calculation cloud diagrams, measurement point displacement, measurement point stress, etc. are all the calculation cloud diagrams exported from COMSOL software, and we think that there is no problem with these diagrams in terms of clarity and graphical standardization.
3. Regarding the lack of clarity in the mathematical modeling section: According to your suggestion, we have reorganized the structure of the article, focusing on the mathematical modeling section to ensure that there is relevance and logic between each part. The specific changes are as follows: the original Chapter 3 (Analysis of engineering examples) is split into two chapters, Chapter 3 (Engineering overview) and Chapter 4 (Numerical modeling). The modified Chapter 3 mainly introduces the basic situation of Dakai Reservoir dam and the arrangement of the dam body monitoring system, while Chapter 4 mainly introduces the process of mathematical modeling, including model selection, control equations, model parameters, and validation of the model after modeling. This change is more in line with the steps of numerical model building and makes the article more clearly organized. Please see revised manuscript with track changes for details.

4. Regarding the validation part and the authenticity of the monitoring data: First of all, we understand your concern about the authenticity of the data, and we only provide the monitoring data at a specific time in the manuscript for two main reasons. One is that the soft soil dam base rheology is time-sensitive, the dam base rheology will evolve over time, and the effect on the dam base rheology is significant when the external action on the dam base changes, so this paper chooses several key periods to compare the simulated and calculated values of the model, such as the period of filling completion, initial impoundment, the first 2-4 years of operation, and operation stabilization. Secondly, Dakai reservoir dam was built for a long time, and it does not have the condition of continuous data monitoring and real-time transmission back, and the collection of monitoring data mainly relies on manual readings to complete, and the data collection is carried out by the operation management personnel holding the readout meter at a certain period of time. In addition, the dam monitoring data belongs to the internal information of the project operation and management unit, we do not have the authority to disclose all the monitoring data, and the measured values appearing in this paper are also disclosed with the consent of the operation and management unit. In order to solve your concerns and troubles, we have added pictures of the monitoring equipment and data acquisition process in the uploaded revised manuscript with track changes (3.4 Monitoring System Layout), and explained the process of monitoring data acquisition, which hopefully can answer your question.

5. Regarding the incomplete discussion section: In response to your suggestion, we have expanded the discussion section, please see lines 515-561 in the revised manuscript with track changes.

We sincerely hope that these changes can solve the problems you raised and improve the quality of the article, we are very grateful for your valuable comments and look forward to your further feedback, if by any other need to improve the place, we are willing to continue to strive to improve.

Reviewer #5:

The quality of this manuscript has been relatively high after two rounds of revisions. And it is to be recognised and appreciated that the innovativeness of this manuscript is very clear, as the authors make it clear in the introduction that existing studies have ignored the effects of rheology. This study proposes a new combined rheological cell model based on the classical combined cell model and also simulates the seepage and displacement of a deep soft soil composite dam foundation using COMSOL software. However I think this manuscript should also address the following minor issues before acceptance:

1.It is suggested that the formula of 122 lines can be numbered.

Response: In response to your suggestion, we have numbered the formulas in line 122 and reordered and renumbered all the formulas that follow, as shown in lines 122-159 of the revised manuscript with track changes.

2.On line 151, 'see in the picture' should be 'see in Fig.3'

Response: In accordance with your suggestion, we have changed “the picture” to “Fig 3” to make the expression more accurate, as shown in line 151 of the revised manuscript with track changes.

3. The "Kpa" in the text should be "kPa", for example, line 187, line 190.

Response: We have checked all the international units in the manuscript and corrected the incorrect writing, and we thank you for these details that helped us avoid unnecessary errors, as shown in lines 190 and 192 of the revised manuscript with track changes.
4. The author is requested to add more detailed information of the project example in "3.1 Summary of project", such as the location of the dam in China, etc. A special picture needs to be drawn, which should show a complete map of China and the location or coordinates of the project.

Response: In response to your suggestion, we have added a picture of the project location in 3.1 Dam Overview, which clearly shows where Dakai reservoir is located in China, as shown on line 174 of the revised manuscript with track changes.

5. In "3.4 Monitoring system layout" of the manuscript, the author shows various kinds of sensors that can be used to monitor the displacement and seepage of DAMS. I am very curious about whether the author installed these sensors 11 years ago, dug up the dam and buried the sensors in it, or how they were installed inside the rock and soil body. It is well known that it is difficult to bury sensors into the interior of a geotechnical body, but the authors seem to have done it easily and buried many different types of sensors. And I'm also wondering, did the author just put the computer and collector directly on the site for 11 years, which doesn't seem realistic, or did he utilise some kind of wireless networked data transmission device to monitor the site remotely? None of this key information and details are mentioned by the authors in the latest version of the manuscript. The author does not describe in detail when the monitoring time started and ended, and the text does not contain any pictures of on-site installation of sensors and equipment. I don't think it's appropriate.

Response: First of all, the clay core wall rockfill dam selected in this paper has been designed with special monitoring design, based on the relevant specifications approved and released by the Ministry of Water Resources of China, according to the project grade, scale, structural type and its topography, geological conditions and geography, etc., to set up the necessary monitoring projects and their corresponding facilities, the specific monitoring system layout and the equipment used are described in the 3.4 part of the manuscript. During the construction of the dam, the installation of the monitoring equipment was carried out as part of the construction process. As the dam is filled, the monitoring equipment is installed inside the dam, and then the signal lines and cables of the monitoring equipment are centrally led out to the observation station at the top of the dam, where subsequent monitoring is carried out. In addition, the monitoring equipment will be protected accordingly before the layering of the dam, which is used to ensure the safety of the equipment. When the monitoring equipment is installed and debugged correctly, the data collection of the displacement and deformation of the dam body, stress and strain, etc., can be carried out, and the monitoring process will continue with the operation of the dam. At present, the equipment and construction process for dam monitoring in China are quite mature, and the monitoring instruments have been developed to the stage of automatically collecting readings and transmitting them in real time, whereas Dakai Reservoir Dam adopts the form of manually collecting readings at regular intervals, and compared with the automated monitoring technology, the traditional and conventional monitoring methods do not have significant innovations, therefore, we did not introduce this part of the manuscript intentionally. Section 3.4 of the revised manuscript with track changes provides an explanation of the monitoring data collection process, which we hope will enable the reader to better understand.

6. According to the description in this paper, these devices are quite advanced, the longest monitoring time can reach 4000 days, the equipment and sensors run continuously for more than 10 years, and the data acquisition instruments are constantly powered up during operation, which is very scary, and I am a little curious about how the author did it. In order to let readers have a better understanding of the equipment, it is best to add a special section detailing the content of the equipment and devices, introducing the models of the equipment and sensors used in the field test of this study, and it is best to put the schematic diagram of the equipment.

Response: As you can see, China has now developed to the stage of intelligent and full life cycle monitoring in dam monitoring, where the monitoring equipment will carry out continuous monitoring work with the operation of the dam. The monitoring equipment in this manuscript does not need to be energized for a long period of time after installation. When we need to collect monitoring data at a certain stage or a certain time, we only need to connect the data collection equipment to the signal line of the monitoring equipment, and readings can be completed through the transmission of signals. Since the monitoring equipment and methods used in this project are not state-of-the-art at present, and the focus of this manuscript is to verify the feasibility of the established rheological coupling model in simulating soft soil composite foundations, there is no dedicated section in the manuscript to introduce the monitoring system of the dam. According to your suggestion, in order to provide the readers with a better understanding, we have explained the process of monitoring data acquisition in 3.4 Monitoring System Layout and added pictures and flowcharts of the monitoring equipment, which are shown in lines 239-250 of the revised manuscript with track changes. The following figure shows a schematic diagram of how the earth pressure gauge works.

Reviewer #7:

This paper establishes a new rheological model of soft soil composite foundations to study the deformation, stress and rheological characteristics of core wall rockfill dam and composite foundations. This topic is interesting and important for engineering practice. However, there are some outstanding issues that must be noted:
1. The papers writing logic is poor and fails to grasp the core description. Keywords are not accurate enough. For example, why is “elemental modeling” used as a keyword? It is suggested that the author should strengthen the revision of paper writing.

Response: You pointed out that our use of “elemental modeling” as a keyword is not accurate enough, we totally agree with you, so we have reevaluated and chosen a more accurate keyword to reflect the research content and focus of the paper. As for the problem of illogicality in the thesis, we did find that the section on numerical modeling was a bit confusing, so we have rearranged and reorganized the logic of the confusing chapters, and split the original Chapter 3 (Analysis of engineering examples) into two chapters, namely Chapter 3 (Engineering overview) and Chapter 4 (Numerical modeling). This change is more in line with the steps of numerical modeling and makes the text more clearly organized. Please see the document “Revised Manuscript with Track Changes” for details.
2. The description of “Soft soil composite foundation rheological modeling” in Part 2 is confused. For example, E2 in formula 4 and S in formula 6 are not explained, and Eα in the later explanation of formula 7 does not appear in the formula, so I do not know what it means.

Response: Thank you very much for pointing out the details in the manuscript. In fact, during the last revision, we have found that some formulas were written with errors and missing symbol meanings, etc. Therefore, we have corrected the errors in the revision and explained the meanings of all the parameters, and added a list of symbols after the conclusions, please see line 592 of the revised manuscript with track changes for details.

3.Many technical terms in Part 3 are used incorrectly, such as clay core wall rockfill dam instead of clay heart wall rockfill dam, and the description of elevation should be used instead of height, unit kPa. Instead of Kpa;

Response: We fully agree with you about the inappropriate use of technical terms that you pointed out. In fact, in the last round of revisions, we rechecked all the technical terms in the text and made changes, see the green highlighted section in the latest upload of revised manuscript with track changes. We have checked all the international units in the manuscript and corrected the incorrect writing, and we thank you for these details that helped us avoid unnecessary errors, as shown in lines 190 and 192 of the revised manuscript with track changes.

4.The horizontal displacement, settlement and stress in Fig.7(b), Fig.8(b) and Fig.10(b) do not conform to the distribution law of deformation and stress during the storage period. It is recommended to check it carefully and modify it.

Response: Figures 7(b), 8(b) and 10(b) should refer to the horizontal displacement contour, settlement contour and stress contour of the dam after the water storage is completed. In fact, in the last revision, we found that the contour plots did not accurately reflect the simulation results, so the original contour plots were replaced by cloud plots in the last uploaded revision (Revised Manuscript with Track Changes), which are shown in Fig. 13-Fig. 16 in the latest manuscript.

Reviewer #8: The authors have made explicit changes and improvements to the paper in response to the reviewers' comments, as detailed below:
1. The authors have provided detailed maps and explanations of the experimental parameters and monitoring point layouts in the revised manuscript, supplemented by high-resolution relevant diagrams.
2. The authors clearly state the innovation of the paper in the introduction section.
3. The authors have optimized the structure of the paper, added a list of symbols and adjusted the title numbering to improve the logic and readability of the paper.
4. The authors have reorganized the content position of the model validation section to enhance the logic of the paper; and added a 2D COMSOL result chart and a model construction flow chart to further clarify the research process.
5. The authors have added a new section on research limitations, pointing out that the existing models do not sufficiently take into account anisotropy and multi-field coupling effects in practical applications, and suggesting directions for future research.
6. The authors have touched up the language of the article and fixes the terminology problem.
The revision of the article responds to the comments made by the reviewers, resulting in a significantly improved paper in terms of structure, content and scientific quality, which meets the requirements for publication, and a small amount of proofreading is recommended.

Response: Thank you very much for your valuable time and effort to review our paper, we are honored by your positive comments from the paper's innovativeness, paper structure, paper readability, etc. Your positive comments and valuable opinions are one of the driving forces for us to continue our scientific research on fluid-structure coupling in soft soil composite foundations, and we will surely proofread the article carefully in order to meet the publication standard in the later stage, and we look forward to your further guidance. We look forward to your further guidance.

Yours sincerely,

Prof. Haitao Mao

Email addresses: maohaitao1234@163.com
