# Supplementary material for: Research on composite foundation rheology using the combined rheological element model
Source: PLoS One. 2025 Jul 10;20(7):e0327560. doi: 10.1371/journal.pone.0327560 (PMC12244545; doi:10.1371/journal.pone.0327560)
Supplement: Attachment — Submitted filename: Response_to_Reviewers_auresp_4.docx [file pone.0327560.s009.docx]

Dear academic editor and reviewers:

Thank you for your letter and the reviews’ comments on our manuscript entitled “Research on composite foundation rheology using the combined rheological element model ” (PONE-D-24-13121).Those comments are very helpful for revising and improving our paper, as well as the important guiding significance to other research. We have studied the comments carefully and made corrections which we hope meet with approval. The main corrections are in the revised manuscript and the responds to the reviews’ comments are as follows.

Replies to the reviewers’ comments:

Reviewer #3:

After several rounds of revisions, the quality of the article has been slightly improved, but there are still some issues. For example, regarding Figure 4, I strongly suggest that the authors refer to more published papers on how to present maps. They can use software such as Google Earth or ArcGIS to present the map of China. Currently, there are a lot of Chinese characters on the figure. It should be noted that this is an international journal. In terms of the elaboration of innovativeness, the comparison with cutting-edge research is not in-depth enough, and the advantages of the new model in international similar research have not been fully highlighted. In addition, there are problems such as poor consistency in the use of professional terms, some expressions that are not concise enough, and incomplete information labeling in the figures, as well as issues with the clarity and aesthetics of the figures that need to be improved.

Response: First of all, I would like to express my sincere appreciation for the time and effort you have invested in reviewing our manuscript. We value the valuable comments you have provided and have carefully considered each piece of feedback.

1.Regarding the issue that Figure 4 does not meet the national journal standards:

Thank you for your constructive suggestions regarding the presentation of Figure 4. We have carefully revised the map according to your feedback: 1) Language Standardization: All Chinese characters in the original figure have been removed and replaced with English labels to align with the international journal standards. 2) Cartographic Compliance: The revised map was regenerated using ArcGIS with strict adherence to China’s mapping regulations and international publishing guidelines.

The updated Figure 4 has been submitted with the revised manuscript. We confirm that all maps comply with both Chinese legal standards and the journal’s cartographic policies. Your attention to detail has significantly improved the quality of our work.

2.Regarding the lack of clarity in innovative articulation: Thank you for your valuable input on highlighting the innovative and international relevance of our work. In the revised manuscript, we strengthen the discussion of rheological models and explicitly address their limitations in terms of long-term settlement prediction and parameter identification challenges. In order to reduce the gap between the existing model and the actual engineering requirements, we propose a hierarchical modeling method with clear mathematical concepts and simple parameter calibration. Unlike previous studies which only focused on consolidation or rheology, our model not only characterized the consolidation and rheology behavior of soft soil, but also considered the seepage and stress coupling of the foundation, which is crucial for the study of deep soft clay foundation. The advantages of this model are verified by the case study of Dakai clay core rockfill dam. Compared with the traditional consolidation model, the settlement prediction error of this model decreases gradually with time and is much lower than that of the consolidation model. We believe that these revisions fully demonstrate the originality and technological advancement of our approach. The above changes have been highlighted in yellow in the introduction to the revised manuscript, specifically on lines 26-29, 53-60 and 69-87.

3. Regarding the poor use of technical terms and the lack of concise language: We sincerely appreciate your valuable feedback on the standardization of terms and the simplicity of language. In response to your comments, we have made systematic improvements throughout the manuscript:

1)Unity of terminology

To ensure professional coherence, we have systematically standardized terminology throughout the manuscript:

①Fluid-solid interaction: Replaced non-standard terms (e.g., "fluid-structure"→"fluid-solid"; "underlyment"→"underlying stratum").

②Engineering terms: Revised structural nomenclature (e.g., "clay core wall rockfill dam"→"clay core rockfill dam"; "core wall"→"clay core"; "repressive platforms"→"toe berm").

③Unit standardization: Abbreviated units in compliance with SI conventions (e.g., "meters"→"m").

④Technical vocabulary: Optimized key terms (e.g., "gravel piles"→"stone columns"; "osmometer"→"piezometer"; "seepage control wall"→"cutoff wall"; "reinforcing layer"→"reinforced zone").

All terminology revisions are highlighted in green in the revised manuscript.

2）Expression Conciseness

We have rigorously streamlined redundant descriptions to enhance clarity:

①Section 2.3 (Lines 158–161) & Section 3.3 (Lines 198–208): Condensed repetitive explanations while preserving technical accuracy.

②Dam description (Lines 176–179): Removed redundant qualifiers and restructured sentences for improved readability.

Optimized sections are marked with light blue highlighting for ease of review.

We believe these revisions have substantially enhanced the manuscript's professionalism and conciseness. Should any further refinements be required, we welcome the reviewer's guidance. Thank you once again for your invaluable feedback.

4.Regarding the issue of image resolution: we have meticulously reviewed all figures in the manuscript and implemented the following revisions: 1)Re-annotated textual elements within the figures (e.g., Figure 6) to ensure clarity and consistency. 2)Updated the annotations in relevant figures (Figures 5 and 7) following the standardization of terminology throughout the text. 3)Addressed instances of unclear numerical representations in Figures 13 to 16 by enhancing their visibility and legibility.

Reviewer #8:

The paper proposes a novel combined rheological element model based on classical composite element modeling and applies it to simulate the rheology-consolidation coupling behavior of deep soft soil composite dam foundations. And the authors have made explicit changes and improvements to the paperin response to the reviewers' comments. However, the following improvements are suggested:
1、The transition between Section 4.3.2 ("Parameter Verification") and Section 5 ("Results Analysis") is abrupt. A brief overview outlining the objectives and framework of subsequent analyses should be added before Section 5.1.

Response:

We sincerely appreciate the reviewer’s insightful comment. In response to the suggestion, we have added a transitional paragraph at the beginning of Section 5 ("Results Analysis"), prior to Section 5.1, to explicitly outline the objectives and framework of the subsequent analyses. The added text includes:

1) A reaffirmation of the model’s reliability based on validation results, emphasizing the consistency between simulations and measurements.

2)A clear statement that this section will systematically investigate the time-dependent evolution of multi-physical fields in the composite foundation under three working conditions, focusing on deformation, stress, and seepage.

3)A declaration of the intent to identify critical thresholds governing long-term stability through quantitative analysis.

This change is intended to enhance the logical connection between chapters and to help the reader grasp the core direction of the subsequent analysis in advance. The added text has been indicated in blue in the revised manuscript, as shown in lines 414-420. We thank the reviewer for their meticulous evaluation and constructive feedback.

2、Contour labels in Figures 13–16 are unclear, adjusting color contrast or adding zoomed-in insets would improve readability. And time axes in Figures 9 and 12 are labeled as "Time(d)" without clarifying "days."

Response:

We appreciate this constructive feedback. The following revisions have been implemented:

1)Label clarity: Enlarge the contour labels in Figures 13 to 16 and adjust its font size according to the background so that readers can see it clearly.

2)Unit ambiguity: We have revised the time axis labels in Figures 9 to 12 to explicitly state the unit as “Time (days)”. Additionally, all units in the manuscript have been standardized to avoid similar ambiguities.

3、The conclusion mentions "scientifically considering rheological effects" but lacks specific engineering measures, practical guidance based on model results should be added.

Response:

We sincerely thank the reviewer for their valuable feedback. In response to the suggestion to include specific engineering measures in the conclusion, we have added a detailed discussion of engineering control methods and practical guidance based on the model results in the final paragraph of Section 6.1. The added measures include:

1) During the foundation treatment phase: extending the pre-loading period of the dam foundation to ensure adequate consolidation of soft soil; installing a three-dimensional drainage system (with longitudinal and transverse configurations) to accelerate the dissipation of pore water pressure; increasing the penetration depth of reinforcement piles to enhance bearing capacity.

2) During the dam filling phase: adopting intermittent staged loading to mitigate rheological risks.

3) During the dam operation phase: implementing long-term settlement monitoring to ensure operational safety.

These measures are directly linked to the model-derived insights on pore pressure evolution, stress redistribution, and time-dependent deformation patterns. The revisions strengthen the translation of theoretical findings into practical engineering applications. The reviewer's suggestion has significantly enhanced the practical value of the conclusions, and we are deeply grateful for their constructive input. Please refer to the red text in the revised manuscript, particularly lines 573–580, for the specific changes.
