# Supplementary material for: Research on composite foundation rheology using the combined rheological element model
Source: PLoS One. 2025 Jul 10;20(7):e0327560. doi: 10.1371/journal.pone.0327560 (PMC12244545; doi:10.1371/journal.pone.0327560)
Supplement: Attachment — Submitted filename: Response_to_Reviewers_auresp_5.docx [file pone.0327560.s010.docx]

Dear academic editor and reviewers:

Thank you for your letter and the reviews’ comments on our manuscript entitled “Research on composite foundation rheology using the combined rheological element model ” (PONE-D-24-13121).Those comments are very helpful for revising and improving our paper, as well as the important guiding significance to other research. We have studied the comments carefully and made corrections which we hope meet with approval. The main corrections are in the revised manuscript and the responds to the reviews’ comments are as follows.

Replies to the reviewers’ comments:

Reviewer #3:

The authors have basically addressed the issues and concerns raised by the reviewers. The decision on acceptance is left to the editors.

Response: Thank you for your constructive feedback and confirmation that our revisions have adequately addressed the concerns raised. We appreciate your professional assessment of our work.

Reviewer #5:

The manuscript has undergone four rounds of revisions and now demonstrates substantial improvement in clarity, technical rigor, and presentation. The proposed combined rheological model effectively addresses the rheology-consolidation-seepage coupling in deep soft soil composite foundations, and the case study validation using COMSOL is well-executed. While the paper is nearly ready for acceptance, minor revisions are required to address lingering issues related to clarity, methodological details, and contextualization within the broader literature.
I believe that once this manuscript is accepted and published, it will be cited by other scholars with a high probability and frequency. I hope the author can continue to carry forward the scientific research spirit of not giving up, not giving up and working hard, and implement the final revision well. The following are some final revision suggestions I put forward:
1. The literature review in the introduction (Lines 24–79) focuses on consolidation and rheological models but lacks explicit references to recent studies on deformation monitoring and seepage analysis in similar geotechnical contexts. For instance, the following works could enrich the discussion:
- https://doi.org/10.1038/s41598-024-57598-7
- https://doi.org/10.1007/s10064-024-03793-9
- https://doi.org/10.1007/s10040-024-02844-5
These academic literatures align with the manuscript’s focus on time-dependent deformation and seepage-stress coupling, and their inclusion would better situate the novelty of the proposed model.
Response: We sincerely appreciate your suggestion to strengthen the literature review with recent advances in deformation monitoring and seepage analysis. As implemented in the revised introduction (Lines 80-86), we have incorporated new references addressing integrated field monitoring and coupled analysis [29,30]. Furthermore, we have also revised the first paragraph of the introduction, emphasizing the impact of rheology on the safety of the dam. For details, please refer to lines 36-42 of the revised manuscript.

2. Section 3.4: Clarify Monitoring Equipment Specifications. The monitoring system (Lines 222–258) mentions instruments like "electromagnetic settlement inclinometer tubes" and "piezometers" but omits critical details such as manufacturer names, model numbers, or measurement accuracies.
For example:
Original text (Lines 229–234): "A mobile inclinometer measures the horizontal displacement..."
Revision suggestion: "Specify the inclinometer model (e.g., *SINCO Digitilt*) and its resolution (e.g., ±0.1 mm/m). "
Response: Thank you for your valuable comments. We have carefully revised the manuscript to clarify the monitoring equipment specifications according to your suggestions. In Section 3.4 of the manuscript, for instruments such as electromagnetic settlement inclinometers, portable inclinometers, fixed inclinometers, soil displacement meters, earth pressure transducers and piezometers, the manufacturer models and resolutions have been added. For details, see the yellow highlighted text in lines 232-247. Meanwhile, we have also modified the grammar and structure of the sentences in Section 3.4.

These revisions have enhanced the repeatability of the monitoring system design and enabled better evaluation of measurement accuracy. We believe that these details are now sufficient to address the technical requirements for describing engineering monitoring instruments.

3. Section 4.4: The numerical modeling section (Lines 327–350) describes mesh generation (Figure 10) but does not validate grid independence. A brief sensitivity analysis (e.g., comparing results for coarse vs. fine meshes) should be added to ensure computational accuracy.
Response: We sincerely thank the reviewers for their valuable suggestions on the verification of grid independence. To solve this problem, we added a comprehensive grid sensitivity analysis in Section 4.4 (lines 348-361) and made the following key enhancements:

1. Comparison of meshes with different resolutions: The model is meshed into three levels: coarse, medium, and fine, and the key areas are refined with emphasis, as shown in Figure 10.

2. Quantitative parameter comparison: Three safety-critical parameters under grid resolution were evaluated, as shown in Table 5;

3. Comparison of computational efficiency: The computational times of the three grids were compared, as shown in Table 5.

Ultimately, all simulations retained a medium grid (4,892 elements) because it achieved engineering accuracy while maintaining computational efficiency.

Your opinions have enhanced the rigor of the calculation and confirmed the reliability of our results. We are grateful to the reviewers for this important improvement of our research.

4. Stress values in Table 7 (Page 34) are reported in MPa, but the text (Line 511) refers to "0.00075mpa/month." Standardize units to either MPa or kPa throughout the manuscript. Authors are asked to read through and check the manuscript carefully to ensure that the units of all parameters are consistent and standardized.
Response: Thank you for your careful review and valuable suggestions. We have standardized the units throughout the manuscript as follows:

1. Unit Consistency:

All stress values in tables and the text are now expressed in kPa, MPa and GPa with SI conventions. Replace “0.00075mpa/month”with “0.00075Mpa/month”. See line 629 for details.

2. Global Verification:

We conducted a full-text check to ensure consistency of units for all parameters（e.g. change KN/m³ to kN/m³ in Table 1 and Table 2). These revisions improve the clarity and professionalism of the manuscript. Please see the highlighted changes in the revised manuscript. We appreciate your attention to this important detail.

5. There are problems with the grammar or sentence structure of some sentences in the manuscript, and I would suggest that the author check the entire text and touch up the language in the body.
For example:
Line 446: "impounding period's growth rate is significantly larger than the impoundment period's..." → "the growth rate during impoundment is significantly higher...".
Line 608: "since it will have an impact" → "as it may adversely affect."

Response:

We sincerely appreciate the reviewer's meticulous attention to linguistic precision. In response to this comment, we have conducted a comprehensive, line-by-line revision of the manuscript with the following actions:

1. Grammar Refinement

The past passive voice describing the general method tense becomes present tense (e.g. , Line 211: replaced "was adopted to..." with "dictates employing...")

2. Terminology Standardization

Ensured unified terminology across all sections (e.g., replaced "permeability coefficient" with "hydraulic conductivity")

3. Elimination of Redundancy

①Removed colloquial expressions (e.g., Line 199: replaced "that can be dug up to ..." with "exhibiting… ")

②Streamlined verbose constructions (e.g., Line 195: condensed "which is loose and highly permabele..." to "loose, highly permeable...")

4. Implementation of Reviewer's Specific Suggestions

①Line 459: Revised to "the growth rate during impoundment being significantly higher than during filling..."

②Line 623: Modified to "as it may adversely affect..."

All modifications have been marked in red in the revised manuscript. We believe these refinements significantly elevate the manuscript's linguistic quality and readiness for publication.

Reviewer #9:

1. DECLINED to be recommended. A draft after 4 rounds of revision doesn’t have keyword???
   Response: Thank you for highlighting this important point. We sincerely apologize for the oversight of not including keywords in the manuscript body.

During initial submission, we strictly followed PLOS ONE’s official formatting sample (formatting_sample_main_body), which did not contain a keywords section. However, we confirm that keywords (Rheology; Rheological and consolidation coupling model; Soft soil composite foundation; Stress deformation; Seepage) were fully entered in the designated field within the online submission system. We acknowledge that keywords should appear in the manuscript for clarity. We will immediately add the following section to the revised manuscript:

**Keywords: Rheology; Rheological and consolidation coupling model; Soft soil composite foundation; Stress deformation; Seepage**

This will be placed below the Abstract in accordance with journal guidelines. We appreciate your vigilance in improving our manuscript’s completeness.
2. Fig 9 denotes the modeling flowchart?? It trivially describes the time dependency, while the draft doesn’t provide how time analysis of data is steps of filling were analyzed!!! On the other hand, data are time dependent and require continuous updating (dynamic database) while this work doesn’t provide a solution for uniformly normalizing dynamic databases via different factors. Search for new approaches via keywords like normalizing large scale sensor-based data with an automated method …
Response: We sincerely appreciate the reviewer's attention to the temporal aspects of our model. It should be clarified that this study belongs to geotechnical rheological process simulation. The temporal dependency originates from physical constraints of the soft soil rheological constitutive relationship and the construction/impoundment schedule, rather than dynamic data stream processing in computer science. We have reinforced this core attribute through the following modifications:

1. The temporal workflow is strictly driven by physical timelines.

①Filling completion period: Incremental loading inherits stress states from previous stages (Prestressed Study), with timesteps defined by the construction schedule (3 months/stage).

②Initial impoundment period: Water level variation follows the physical function

h(t) = 75 + 0.5t (m) (0.5 m/day), completing impoundment in 36 days.

③Operational stabilization period: Steady-state analysis covers 10 years of physical time.

2. Physical time progression is explicitly handled by COMSOL's Time-Dependent Solver, with inter-stage field variable transfer via boundary condition inheritance (no database updating required).

①We have explicitly visualized the physical timeline in the revised Fig. 9: Added stage duration labels (e.g., 3 months)

②Annotated the impoundment function h(t)

③ Indicated physical progression direction with timeline arrows .

3. Inapplicability of Specific Terminology:

The mentioned concepts (e.g., automated normalization, dynamic data updating) are not applicable to our physics-based model.

①"Dynamic Updating" manifests as inheritance of physical field variables, not database operations.

②The core innovation lies in the composite rheological unit model, not data algorithms (see Abstract and Section 2).

These modifications further clarify that temporal dimensionality in this study is an inherent attribute of the physical process, not an independent data processing workflow. We thank the reviewer for prompting us to present this key aspect more clearly.

3. Lack of convincing information about the used assumptions? Type of mesh and why? The boundary condition and its definition? Used data for model construction and the method of incorporation?
Response: We thank the reviewer for raising these critical methodological aspects. Detailed information is already provided in our manuscript, and we clarify each point below with specific cross-references:

1. Key Assumptions:

①Unsaturated→saturated transition: Richards' equation

②Saturated foundation: Poroelastic theory

③ Material models: Composite rheology (soft soil), Duncan E-v (rockfill), Linear elasticity (cutoff wall).

2. Mesh Selection Rationale :

①Type: Locally refined triangle meshes.

②Reason: Medium mesh (4,892 elements) optimized accuracy (vs. fine mesh: <2% error in critical displacements/stresses) and computational efficiency.

3. Boundary Conditions :

①Hydraulic: Reservoir h(t)=75+0.5t (m); Foundation base: zero pressure.

②Mechanical: Foundation sides - roller supports; Base - fixed.

③Initial: Geostatic balance + staged stress inheritance.

4. Data Integration:

①Parameters from: field monitoring + inverse analysis for rheology.

②Validation: inversion parameter vs. experimental parameter (Table 3).

③Implementation: COMSOL material modules + Prestressed Study.

These elements collectively ensure the model’s fidelity to real-world dam behavior.
4. Considering the SSI???
Response: Thank you for highlighting this fundamental aspect. Soil-structure interaction (SSI) is intrinsically embedded in our modeling framework through three interconnected mechanisms. First, mechanical coupling is achieved via the proposed composite rheological unit (Section 2), which explicitly integrates dam structure and soft soil foundation behavior. This enables stress transfer at interfaces through staged loading simulation and nonlinear rockfill-soil interaction resolution (Duncan E-v model in Section 4.3.1). Second, hydromechanical coupling captures the closed-loop SSI: reservoir impoundment induces pore pressure changes → alters effective stresses → drives foundation deformation → feeds back to dam stability. This process is quantified using Richards' equation for unsaturated-saturated transition and Biot's consolidation theory (Eq. 40, Section 4.2.1) for saturated conditions. Third, long-term SSI effects manifest through continuous stress redistribution between the dam and foundation via soft soil creep, driving post-construction settlement and decadal-scale stress evolution. In essence, SSI forms the operational core of our coupled model rather than functioning as an add-on component.

5. The given FEM model definitely is incorporated with truncation and round off errors. How did you evaluate these errors and with what criteria??
Response: We appreciate this insightful technical query regarding numerical errors.

Our error management strategy aligns with standard FEM engineering practices:

1. Truncation Error Evaluation:

Spatial discretization error was quantified via mesh convergence tests (Coarse/Medium/Fine meshes in Table 5). Maximum variation in critical outputs (displacements/settlements/stresses) was <2% between mesh levels–well below the 5% threshold accepted in geotechnics.

2. Round-off Error Control:

COMSOL's default double-precision solverreduced single-operation rounding errors to 10^-16^. The global stiffness matrix condition number remained <10^4^ , preventing significant error amplification.

These protocols ensure numerical uncertainties are negligible relative to the model's predictive capability .
6. COMSOL only analyzes the nonlinearity of the model, not the actual structure. Since the response of a structure is sensitive to the strengths and stiffnesses of its components, the actual properties may not be known accurately. Capacity design can greatly reduce uncertainty. No information regarding these problems and treatment methods has been given.
Response: We appreciate the reviewer's emphasis on structural nonlinearity and uncertainty management. While capacity design is essential for seismic structures, our study focuses on validating the composite rheological model under controlled geotechnical processes. To address material property uncertainties, three key measures were implemented:

1.Rheological parameters were determined via inverse analysis, ensuring fidelity to actual soft soil behavior;

2.Material nonlinearity was captured using the Duncan E-v model;

3.Local mesh refinement at critical zones (clay core and cutoff walls) resolved stress concentrations.

Collectively, these protocols enable the model to replicate observed field responses within 5% error, establishing its reliability for static geotechnical scenarios beyond seismic capacity design paradigms.

7.Discussion should be documented via technical limitations/physical interpretation of the results/impact of bias of the used data on the results/real practical and evidential analysis/considering the effect of subsurface spatial distribution of soil/rock types; the effect of clay sensitivity; involved uncertainty for the post processing geo-model (may get good hints from https://link.springer.com/article/10.1007/s10064-018-1400-9, https://www.sciencedirect.com/science/article/abs/pii/S0013795224002655, https://www.mdpi.com/2220-9964/10/5/341, https://link.springer.com/article/10.1007/s10064-018-1400-9, https://www.sciencedirect.com/science/article/abs/pii/S0013795215000411, https://www.sciencedirect.com/science/article/pii/S0341816222002752, https://link.springer.com/article/10.1007/s00366-023-01852-5, …)/computational time and cost/solid comparison with other scholars and models…
Response: We sincerely appreciate the reviewer's insightful suggestions to strengthen the Discussion section. As requested, we have explicitly addressed the following key aspects by incorporating targeted enhancements:

1. Spatial heterogeneity & clay sensitivity effects:

Added: "The spatial heterogeneity of subsurface soil/rock types, particularly the distribution of sensitive clays known to reduce shear strength, may exacerbate localized rheological deformation [44,45]." This directly responds to the concern on subsurface spatial distribution and clay sensitivity impact.

2. Model uncertainty quantification:

Added: "However, uncertainty in predicting the precise spatiotemporal magnitude of rheological effects remains a challenge; advanced ensemble-based deep learning for subsurface modeling shows promise in future uncertainty quantification [46]. " This acknowledges limitations in geo-model uncertainty (supported by recommended literature insights).

3. Data bias mitigation:

Added: "Initial parametrization could be enhanced via multi-source data fusion techniques to better capture subsurface heterogeneity and reduce dependency on sparse monitoring points [47]." This confronts potential data bias and aligns with literature-prompted solutions.

These additions enrich the physical interpretation, uncertainty awareness , and practical strategies against data bias, while maintaining the original focus on rheology-driven mechanisms. We believe this revision holistically addresses the reviewer's concerns.

8.English revision with the help of a native agent
Response: We sincerely thank the reviewer for their valuable suggestion regarding the English language quality of our manuscript. We agree that clear and precise language is essential for effective communication of our research. In direct response to this comment, we have conducted a thorough revision of the entire manuscript to improve its English expression. Specifically, this revision addressed:

1.Inconsistent terminology: We have standardized all key terms throughout the paper.

2.Grammatical inaccuracies: We have carefully reviewed and corrected grammatical errors.

3.Redundant phrasing and awkward sentence structures: We have streamlined sentences for better clarity and conciseness.

To facilitate the reviewer's examination of these changes, all modifications made during this comprehensive English revision have been clearly highlighted in RED FONT within the revised manuscript document. We believe these revisions have significantly enhanced the clarity and professionalism of the manuscript. We are grateful for the reviewer's time and insightful feedback, which has undoubtedly strengthened our paper.

9. Verification of the model is not certified. It doesn’t provide solid comparative analysis
Response: We acknowledge the reviewer's concern regarding limited validation data. The manuscript presents monitoring data only at specific time points due to two primary constraints: First, the time-dependent rheology of soft soil foundations exhibits continuous evolution, with external load variations significantly influencing rheological behavior. Therefore, key operational stages—including filling completion, initial impoundment, early operation (2–4 years), and stabilized operation—were selected for model validation to capture critical rheological phases. Second, the Dakai Reservoir dam, constructed decades ago, lacks continuous automated monitoring infrastructure; data collection relies on periodic manual readings by operational personnel. Additionally, comprehensive monitoring datasets are classified as internal assets by dam operators. The published measurements represent authorized disclosures, and regrettably, continuous data cannot be provided.

To address the depth of comparative analysis, we have significantly strengthened the interpretation of validation results in the revised manuscript (lines 384–391 and lines 421–431). This includes: 1) Statistical error analysis across all monitoring points and operational phases; 2) Identification of root causes for observed discrepancies; 3) Explicit linkage between anomalies and the rheological mechanisms proposed in our model.

10. Improper captions. For example, Figs like 11 have different parts but none of them are reflected in the caption.

Response: We sincerely appreciate your feedback on improving figure captions. All multi-panel figures (Figs. 11, 12) have been checked to explicitly specify the content of each subfigure in their captions.

For example, the updated caption of Fig. 11 and 12 now reads:

Fig 11. Time-history displacement curves at monitoring points: (a) Horizontal displacement at DK1; (b) Horizontal displacement at DK2; (c) Settlement at SW1; (d) Settlement at SW2.

Fig 12. Stress-time curve at monitoring points: (a)Stress at SG1; (b)Stress at SG2.

This ensures readers can immediately associate each subplot with its corresponding dataset without referring to external text. In addition, we have revised Figures 5 and 6 to provide clearer annotations and enhanced hierarchical organization within the illustrations.
